# Transcriptomic and metabolomic reveals silicon enhances adaptation of rice under dry cultivation by improving flavonoid biosynthesis, osmoregulation, and photosynthesis

**DOI:** 10.3389/fpls.2022.967537

**Published:** 2022-08-04

**Authors:** Hao Jiang, Ze Song, Qing-Wang Su, Zhi-Heng Wei, Wan-Chun Li, Zi-Xian Jiang, Ping Tian, Zhen-Hui Wang, Xue Yang, Mei-Ying Yang, Xiao-Shuang Wei, Zhi-Hai Wu

**Affiliations:** ^1^College of Agronomy, Jilin Agricultural University, Changchun, China; ^2^College of Life Sciences, Jilin Agricultural University, Changchun, China; ^3^National Crop Variety Approval and Characteristic Identification Station, Jilin Agricultural University, Changchun, China

**Keywords:** rice, dry cultivation, transcriptomic, quasi-targeted metabolomics, photosynthesis, osmoregulatory, flavonoid metabolism

## Abstract

Dry cultivation is a new rice crop mode used to alleviate water shortage and develop water-saving agriculture. There is obvious genetic difference compared with drought-tolerant rice. Silicon (Si) plays an important role in plant adaptation to adverse environmental conditions and can significantly improve the drought tolerance and yield of rice. However, the regulatory mechanism via which Si provides plant tolerance or adaptation under dry cultivation is not well understood. The present study investigated the changes in plant growth, photosynthetic gas exchange, and oxidative stress of the rice cultivar “Suijing 18” under dry cultivation. Si improved photosynthetic performance and antioxidant enzyme activity and subsequently reduced lipid peroxidation of rice seedlings, promoted LAI and promoted leaf growth under dry cultivation. Further, transcriptomics combined with quasi-targeted metabolomics detected 1416 and 520 differentially expressed genes (DEGs), 38 and 41 differentially accumulated metabolites (DAMs) in the rice leaves and roots, respectively. Among them, 13 DEGs were involved in flavonoid biosynthesis, promoting the accumulation of flavonoids, anthocyanins, and flavonols in the roots and leaves of rice under dry cultivation. Meanwhile, 14 DEGs were involved in photosynthesis, promoting photosystem I and photosystem II responses, increasing the abundance of metabolites in leaves. On the other hand, 24 DAMs were identified involved in osmoregulatory processes, significantly increasing amino acids and carbohydrates and their derivatives in roots. These results provide new insight into the role of Si in alleviating to adverse environmental, Si enhanced the accumulation of flavonoids and osmoregulatory metabolites, thereby alleviating drought effect on the roots. On the other hand, improving dehydration resistance of leaves, guaranteeing normal photosynthesis and downward transport of organic matter. In conclusion, Si promoted the coordinated action between the above-ground and below-ground plant parts, improved the root/shoot ratio (R/S) of rice and increased the sugar content and enhancing rice adaptability under dry cultivation conditions. The establishment of the system for increasing the yield of rice under dry cultivation provides theoretical and technical support thereby promoting the rapid development of rice in Northeast China, and ensuring national food security.

## Introduction

The increasing environmental stress and irrigation water scarcity had been posing serious threats to global rice production (Garg et al., 2003). The International Food Policy Research Institute (IFPRI) study predicted a 15% decrease in irrigated rice yields by 2050 in developing countries due to climate change. Therefore, there is an urgent need to balance the contradiction between food demand and ecological security. Rice under dry cultivation is a mode wherein rice is directly sown under dryland preparation without nursery and transplantation. Here, the whole reproductive period of rice relies on natural precipitation. Moreover, adequate water is resupplied only during critical growth periods or drought. Rice under dry cultivation has been carried out and is rapidly developing worldwide to develop water-saving agriculture and a light and simplified production measure (Jiang et al., 2021). The development of rice under dry cultivation mode can alleviate the water shortage crisis, reduce greenhouse gas emissions and reduce agricultural surface pollution (Adekoya et al., 2014; Weller et al., 2016). However, there is significant genetic (Xia et al., 2014) and epigenetic difference (Luo, 2010; Xia et al., 2016) compared to rice in terms of drought tolerance. Therefore, it is important to explore the mechanisms of plant response to environmental stresses under the dry crop model to improve the adaptability and application of rice.

It is important that crops maintain their normal metabolic processes for long during drought stress (Hu and Xiong, 2014). Maintaining photosynthetic capacity under drought conditions is an important feature of drought-resistant crops (Ma X. et al., 2016). Mumtaz et al. (2020) showed that drought inhibits rice growth by reducing photosynthetic capacity, reducing grain yield and quality. Photosynthetic capacity lowers during drought due to reduced available CO_2_ by stomatal closure or reactive oxygen species (ROS)-induced photooxidative damage under dehydration (Chaves et al., 2009; Saibo et al., 2009). However, many drought-tolerant mechanisms, such as regulation of osmotic pressure through osmolytes, protective metabolites, proteins, and ROS scavenging systems, maintain normal photosynthesis under dehydrated conditions, leading to relatively high biomass accumulation and yield (Valliyodan and Nguyen, 2006). At the cellular level, drought signaling promotes stress-protectant metabolites such as proline and trehalose, triggers the antioxidant system to maintain redox homeostasis, and deploys peroxidase enzymes to prevent acute cellular damage and membrane integrity. Various other factors such as the extent of water stress and the plant organ sensing the stress also trigger specific signaling responses, including, but not limited to, abscisic acid, brassinosteroid, and ethylene phytohormone pathways (Gupta et al., 2020). Plant metabolic responses under drought stress are currently attracting more attention because many metabolites are thought to play a central role in stress tolerance. Moreover, metabolites are better targets for improving drought tolerance than the individual genes as they are generated by the interaction of various genes and pathways, which lead to systemic effects in response to stress (Guo et al., 2018). Ma X. et al. (2016) found that 4-hydroxycinnamic acid and ferulic acid were significantly correlated with drought tolerance in two rice cultivars, and the DEGs involved in the pathways of these metabolites were proposed as good candidate genes to improve drought-tolerance (Ma X. et al., 2016). Meanwhile, Siriwach et al. (2020) examined the metabolic pathways of Arabidopsis thaliana during stress response based on silicon analysis and identified the responses and metabolites related to drought adaptation (Siriwach et al., 2020).

Silicon is the second most abundant element in soil, which exists in almost all plants. It is beneficial to plant growth, especially under biotic and abiotic stress conditions (Huang and Ma, 2020; Ranjan et al., 2021; Huang et al., 2022; Thorne et al., 2022). Studies have shown that Si application improves growth under drought, heavy metals, and salinity in rice, barley, tomato, cucumber, and other crops (Ma, 2004; Ma and Yamaji, 2006; Liang et al., 2007; Zhu and Gong, 2013; Luyckx et al., 2017; Réthoré et al., 2020). Yin et al. (2016) showed that Si upregulated several key phytohormone synthetic genes under salt stress, which also identified the phytohormone involved in Si-induced salt tolerance in sorghum and provided evidence for the role of Si in mediating salt tolerance (Yin et al., 2016). Si also increased root water uptake, regulated phytohormone levels, and increased antioxidant defenses in cucumber under salt stress (Zhu et al., 2019). Recently, Coskun et al. (2019) summarized the roles of Si in higher plants and proposed the “apoplastic obstruction hypothesis” based on molecular and phenotypic data. This model argues for a fundamental role of Si as an extracellular prophylactic agent against biotic and abiotic stresses (as opposed to an active cellular agent), with important cascading effects on plant form and function (Coskun et al., 2019). Several studies have shown that Si induces differential gene expression in response to biotic and abiotic stresses. Chain et al. (2009) analyzed the effect of Si on the transcriptomic changes in wheat under powdery mildew stress and found that Si supply nearly reversed the gene expressions under pathogen attack (Chain et al., 2009). Holz et al. (2015) identified 1136 DEGs induced by supplemental sodium silicate in *in vitro*-generated cucumber clone (Holz et al., 2015). Rasoolizadeh et al. (2018) observed that Silicon protects soybean plants against *Phytophthora sojae*, with the addition of Si, the plant transcriptome remains unaffected under pathogen stress, the expression of effector-coding genes by the pathogen is reduced (Rasoolizadeh et al., 2018). Yoo et al. (2019) compared rice plants treated with *Paenibacillus yonginensis* DCY84T (DCY84T) and Si with untreated rice using RNA-seq, under Si treatment, 576 genes were upregulated and 394 genes were downregulated, and the treatment improved initial seedling growth and enhanced resistance to environmental stresses (Yoo et al., 2019). However, under abiotic stresses, Haddad et al. (2019) studied the transcriptomics of *Brassica napus* roots in response to Si supply and found differential expression of genes involved in plant hormone metabolism and stress response (Haddad et al., 2019). Meanwhile, Zhu et al. (2019) sequenced the transcriptome of control and salt-stressed cucumber leaves in the presence and absence of Si addition. They reported changes in the expression of 1469 genes in response to Si treatment, these genes were mainly involved in hormone and signal transduction and stress and defense responses. Under salt stress, Si treatment shifted the transcriptome of stressed cucumber back to that of the control (Zhu et al., 2019).

Drought and its relationship with silicon have been well studied. For example, in some plants such as sugarcane (*Saccharum officinarum* L.), wheat (*Triticum aestivum* L.) under drought stress, silicon increases ascorbate peroxidase activity, total soluble sugars content, relative water content (Mavrič Čermelj et al., 2021). In some studies, on rice (*Oryza sativa* L.) was found in Si induced different responses regarding to proline accumulation which could be beneficial for drought exposed plants (Mauad et al., 2016). Si might also trigger the transcription of genes that are related to antioxidant defense, photosynthesis, osmotic adjustment, lignin, and suberin metabolism (Zhu and Gong, 2013). However, the effects of silicon on rice under dry cultivation has not yet been studied in detail. Typically, the yield and quality of rice under dry cultivation can be affected due to the changes in cultivation patterns. Our preliminary analysis showed that the optimal silicon application rate suitable for high yield in Jilin Province ranged from 30 to 47.68 kg⋅ha^–1^, resulting in 38–47% yield increase in rice under dry cultivation (Su et al., 2022). The present study analyzed the transcriptome and metabolome of rice treated with Si under dry cultivation. Further, a correlation analysis combined with morphological and physiological trait analysis was performed to identify the specific metabolites, genes, metabolic pathways, and their interactions. The study thus comprehensively evaluates the regulatory role and mechanism of Si under dry cultivation, reveals the molecular mechanism of Si to enhance the adaptability of dry farming rice, and lay the foundation for the sustainable development of the dry cultivation model.

## Materials and methods

### Experiment setup and materials

The experiment was conducted at the National Crop Variety Approval and Characterization Station at the Jilin Agricultural University of Changchun City, Jilin Province (125°39 E and 44°46 N). The soil of this region has an organic matter content of 18.7 g⋅kg^–1^, alkaline dissolved nitrogen of 117.02 mg⋅kg^–1^, available phosphorus of 41.11 mg⋅kg^–1^, available potassium of 245.16 mg⋅kg^–1^, available silicon of 113.46 mg⋅kg^–1^, and pH of 6.2.

The high-yielding rice cultivar “Suijing 18” (China Rice Data Center, No. 2014021)^1^ was used as the test material in this study. Preliminary analysis revealed that “Suijing 18” is a high-quality drought-resistant cultivar suitable for dry farming in the central region of Jilin Province.

The experiment was conducted in pots with rice grown under four treatments of flooding cultivation (W), flooding cultivation with Si treatment (WS), dry cultivation (D), and dry cultivation with Si treatment (DS); Sow five holes in each pot and eight seeds in each hole. The environment of the test is greenhouse, and the temperature is maintained at 25 ± 1°C, the humidity is 80%, and the light/dark cycle of 12 h/12 h is carried out during the whole growth process. Triplicates were maintained per treatment. Biori Russian mineral silica with an effective silica content of ≥72% was applied as a base fertilizer at 45 kg⋅ha^–1^. Calcium superphosphate (P_2_O_5_; 12%) at the rate of 75 kg⋅ha^–1^ and potassium chloride (K_2_O; 60%) at the rate of 75 kg⋅ha^–1^ were applied as a one-time substrate, and urea (pure N; 46%) at the rate of 160 kg⋅ha^–1^ was applied three times as basal fertilizer, tiller fertilizer, and spike fertilizer (5:3:2). The indexes involved in the experiment were measured by collecting samples 45 days after sowing.

### Evaluation of physiological indexes

#### Root system index

The root samples were collected, and the root volume was measured following the drainage method (Gong et al., 2014), The length of the longest root and the number of roots per hole were measured. Three repetitions per treatment were used for the measurements, and the average value was calculated.

#### Leaf photosynthetic parameter

The Li-6400XT photosynthesizer (LI-6400, LI-COR, Inc., Lincoln, NE, United States) was used to measure the photosynthetic parameters with a built-in fixed light source and a light quantum density setting of 1200 μmol⋅m^–2^⋅s^–1^. Five representative plants per treatment were selected for the analysis. The photosynthetic rate was measured between 9:00 and 11:30 in the morning on a clear and windless day, with three replicates per treatment. The mean values were calculated (Wu et al., 2022).

#### Superoxide anion, hydrogen peroxide, and malondialdehyde content

The superoxide anion (O_2_^–^) production was determined by the method described by Sadeghi et al. (2020). The concentration of hydrogen peroxide (H_2_O_2_) was determined according to Brennan’s method (Brennan and Frenkel, 1977). Malondialdehyde (MDA) content in the first unfolded leaf at the upper part of the plant was determined following the thiobarbituric acid method (Zheng et al., 2020). Each experiment was independently repeated thrice.

#### Antioxidant enzyme activity

About 0.2 g of seedling stage frozen plant tissue was homogenized in 5 mL of extraction buffer (100 mmol/L phosphate buffer, pH 7.00). The homogenate was centrifuged at 10,000 × *g* at 4°C for 10 min, and the supernatant was used to determine SOD, CAT, and POD activities. The SOD activity was determined following the nitrogen blue tetrazolium method, POD activity following the guaiacol method, and the CAT activity following the ultraviolet absorption method (Ling et al., 2020), replicated three times measurement, and the mean values were calculated.

#### Leaf area index

Five plants of uniform growth were selected for each treatment, and the total leaf area of all the leaves of a plant was determined using a leaf area meter (CID-203, produced by CID company, WA, United States). The measurements were repeated three times, and the average value was calculated. The leaf area per square meter (leaf area index; LAI) was calculated based on the number of plants per square meter.

#### Leaf relative water content and electrical conductivity

Water retention in the leaves was determined following the method by Clarke (Clarke and Mccaig, 1982). Fresh leaves were collected from each treatment, rinsed with deionized water, and wiped with blotting paper; then, the fresh weight (a) was recorded. The sample was immersed in 28°C distilled water for 24 h, and the turgid weight (b) was determined. The sample was then dried at 85°C for 24 h, and the dry weight (c) was recorded. The water retention rate of the isolated leaves was calculated as follows (b−c/a−c) × 100%

Electrical conductivity was measured based on the method by Sairam and Srivastava (2002). Use portable conductivity meter (DDBJ-305, produced by Shanghai Precision Scientific Instruments Co., Ltd., Shanghai, China). Approximately 0.5 g of leaf tissue was collected, washed, placed into three centrifuge tubes containing 10 mL of ultra-pure water and maintained in a shaking incubator (25°C, 150 rpm) for 2 h. The initial conductivity (E1) was measured, and the conductivity (E0) of the blank control deionized water was also measured, then, the sample was boiled in water for 30 min and cooled the room temperature, then, measuring the electrical conductivity (E2).

#### Root/shoot ratio

The root and shoot samples were dried in an oven at 105°C for 30 min and drying at 85°C to constant weight, then weighed to determine the root shoot ratio. Each treatment was repeated three times, and the average value was calculated.

#### Sugar content

According to the method by Ma et al. (2021), 0.02 g fresh weight of plant tissues were boiled with 800 μL of 80% ethanol in an 80°C water bath for 30 min. The resulting extract was collected to measure the soluble sugar, sucrose, and fructose content using the sulfuric acid anthrone colorimetric method with slight modifications (Ma et al., 2021).

#### RNA-seq

The transcriptomes of rice under dry cultivation with and without Si treatment were generated through the high throughput RNA sequencing. Leaves and roots of fully expanded were collected 45 days after sowing, each sample was frozen in liquid nitrogen with three biological replicates. Total RNA was extracted from samples using TRIzol reagent. RNA integrity was assessed using the RNA Nano 6000 Assay Kit of the Bioanalyzer 2100 system (Agilent Technologies Inc., Santa Clara, CA, United States). The cDNA library was sequenced on the Illumina sequencing was performed at the Beijing Novogene Bioinformatics Technology Co., Ltd. (Beijing, China).^2^ Index of the reference genome was built using Hisat2 v2.0.5 and paired-end clean reads were aligned to the reference genome using Hisat2 v2.0.5. featureCounts v1.5.0-p3 was used to count the reads numbers mapped to each gene. And then FPKM of each gene was calculated based on the length of the gene and reads count mapped to this gene (For DESeq2 with biological replicates). Differential expression analysis of two conditions/groups (two biological replicates per condition) was performed using the DESeq2 R package (1.20.0). Gene Ontology (GO) and Kyoto Encyclopedia of Genes and Genomes (KEGG) tools were used to analyze the DEGs.

#### Quasi-targeted metabolomics

The above-mentioned samples were also used for the metabolome analysis. LC-MS/MS analyses were performed using an ExionLC™ AD system (SCIEX) coupled with a QTRAP^®^ 6500+ mass spectrometer (SCIEX) in Novogene Co., Ltd. (Beijing, China). The detection of the experimental samples using MRM (Multiple Reaction Monitoring) were based on novogene in-house database, Following their standard procedures extraction metabolites, These metabolites were annotated using the KEGG database,^3^ HMDB database,^4^ and Lipidmaps database.^5^ Principal component analysis (PCA) and (orthogonal) partial least-squares discriminant analysis [(O)PLS-DA] were carried out to visualize the metabolic alterations among the experimental groups after mean centering and unit variance scaling. The variable importance in the projection (VIP) was used to rank the overall contribution of each variable to the (O)PLS-DA model; variables with VIP > 1.0 were considered relevant for group discrimination. The metabolites with both multivariate and univariate statistical significance (VIP > 1.0 and *p* < 0.05) were considered as differentially accumulated metabolites (DAMs). Principal component analysis was conducted to visualize the impact of Si on the dry farming rice metabolome.

#### Co-expression network analysis of the metabolome and transcriptome

The Metware Cloud website^6^ was used to assess the correlation between metabolome and transcriptome. Firstly, log2 conversion was performed on the data uniformly before analysis. For the joint analysis between metabolome and transcriptome, the Pearson Correlation Coefficient (PCC) and the corresponding *P*-value were used for screening, and the screening criteria were set at PCC > 0.8.

#### Reverse transcription and gene quantitative real-time PCR analysis

Leaf or root samples were collected during the seeding stage, immediately frozen in liquid nitrogen and stored at −80°C for future use. RNA was extracted using TRIZOL RNA extraction reagent following the manufacturer’s procedure. The first-strand cDNA was reverse transcribed from 1 μg of the total RNA using the PrimeScript™ RT reagent Kit (TaKaRa, RR047A, supplied by TAKARA in Japan). The quantitative real-time PCR (qRT-PCR) was carried out on a Light Cycler 480 Real-Time PCR System (Roche, Germany). Specific primers for each candidate gene were designed using Primer 5 software (Premier Inc., Canada). The qRT-PCR reactions were conducted with three technical replications in a 20 μL reaction volume using the TB Green Premix Ex Taq II (TaKaRa, RR820A) (SYBR Green). The relative gene expression was calculated using the 2^–ΔΔCt^ method (Livak and Schmittgen, 2001).

### Statistical analysis

All experiments were conducted in triplicate (*n* = 3). One-way analysis of variance (ANOVA) and statistical analysis was performed to determine statistically significant differences between the control and other treatments using SPSS 20.0 software (SPSS Inc., Chicago, IL, United States); differences were considered statistically significant at *P* < 0.05. Data are presented as the mean ± standard deviation (SD) of three replicates.

## Results

### Influence of Si on the physiological response of rice under dry cultivation

**FIGURE 1:**
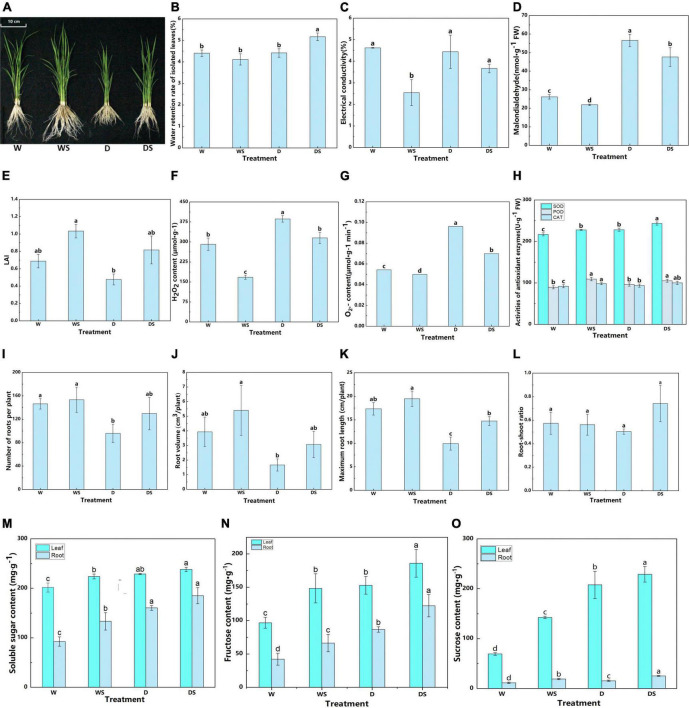
Effect of silicon application on rice under two cultivation conditions. Phenotypes of rice with and without Si under flooding and dry cultivation conditions **(A)**; Relative water retention rate **(B)**, electrical conductivity **(C)**, malondialdehyde (MDA) content **(D)**, leaf area index **(E)**, H_2_O_2_ content **(F)**, O_2_^–^ content **(G)**, and antioxidant enzyme activity **(H)**, root number **(I)**, root volume **(J)**, root length **(K)**, root to shoot ratio **(L)**, soluble sugar content **(M)**, fructose content **(N)**, and sucrose content **(O)** of rice. The panels **(H,M–O)**, different lowercase letters indicate unified indicators significant differences between different treatments. Each experiment was repeated three times. Data represented are the means ± SD of three biological replicates. Different lowercase letters indicate significant differences (*P* < 0.05). W: Rice under flooding cultivation, WS: Rice under flooding cultivation with Si treatment, D: Rice under dry cultivation, DS: Rice under dry cultivation with Si treatment.

First, plant phenotype were investigated to examine the effect of Si on the growth of dry farming rice. In this study, plant growth actually increased significantly after Si treatment under both dry and flooded cultivation conditions. The length in the Si-treated rice was significantly longer than that no Si-treated rice (Figure 1A). Si also has a certain effect on the physiological characters of rice under dry cultivation, as shown in Figure 1B, the relative water retention rate of the leaves in the Si-treated rice was higher than in other treatments. Thus, Si treatment maintained the water-holding capacity of the isolated leaves under dry cultivation. In addition, an indicator of plant lipid peroxidation, MDA can adversely affect membrane integrity under oxidative stress. Relative electrical conductivity is another important index used to evaluate membrane integrity and instability under oxidative stress. Si treatment significantly reduced the MDA content and electrical conductivity compared to those without Si application, both under flooding and dry cultivation (Figures 1C,D). Meanwhile, LAI of rice decreased by 30.81% after dry cultivation, and Si application promoted LAI by 71.28% compared with the dry cultivation (Figure 1E).

At the physiological level, silicon application performed better than no silicon application (Figures 1F–H). The H_2_O_2_ and O_2_^–^ content and the SOD, POD, and CAT activities were affected under dry cultivation compared with flooding. The reactive oxygen species (ROS) such as O_2_^–^ and H_2_O_2_ decreased under Si treatment (Figures 1F,G). The enzyme activities slightly increased, with differences in the extent of the increase. Silicon application increased the SOD, POD, and CAT activities (Figure 1H), which indicates that silicon application help rice to cope with oxidative stress. Furthermore, Si application increased the root length (*P* < 0.05) of rice compared with no Si treatment under both conditions, a similar trend was observed for root volume and numbers (Figures 1I–K). Under dry farming, the root branch density was significantly reduced; however, Si treatment increased the tissue density. These observations indicate that the root growth parameters restricted under dry farming are retained by Si treatment. In addition, Si application improved the root/shoot ratio (R/S) of rice (Figure 1L), with an obvious effect under dry cultivation.

Sugar is a source of energy and an important osmotic regulator. In this study, Si application increased the sugar content under flooding and dry farming conditions (Figures 1M–O). The soluble sugar, sucrose, and fructose content increased by 4.39, 21.57, and 10.10%, respectively in leaves and 15.63, 45.88%, and 60.36, respectively in roots after Si application compared with no Si in dry cultivation. These observations indicate a more significant effect of Si on the sugar content in the roots of rice under dry cultivation.

### Influence of silicon on photosynthetic capacity of rice under dry cultivation

Furthermore, compared with the rice under flooding cultivation, the net photosynthetic rate of dry farming rice from 17.13 μmol m^–2^ s^–1^ decreased to 11.54 μmol m^–2^ s^–1^. The stomatal conductivity (Gs) showed a decreasing trend after drought cultivation. The decrease of photosynthetic rate may be due to the increase of stomatal limitation. However, Si application increased the net photosynthetic rate of rice under dry cultivation (Table 1). Compared with flooding cultivation, leaf intercellular CO_2_ concentration (Ci) showed an increase of 17.18% after dry cultivation, but Ci decreased after Si application, indicating that Si can increase the carbon assimilation capacity of photosynthesis. The present study also found that net photosynthesis rate (Pn) was significantly and positively correlated with AMC (Pn/Ci), suggesting that AMC is more useful in rice research. In addition, Si application increased transpiration rate (Tr) of rice under dry cultivation, while it reduced the water-use efficiency (WUE) (Pn/Tr) of the leaves. The WUE of plants increased significantly and reached significant levels after Si application under both flooding and drought conditions. These observations indicate that appropriate Si levels can reduce the decline of WUE under drought cultivation, and a higher WUE can ensure better photosynthesis of rice leaves.

**TABLE 1:** Effects of silicon on the leaf photosynthetic parameters of rice under flooding and dry cultivation.

Treatment	Pn (μmol CO_2_ m^–2^ s^–1^)	Gs (mol H_2_O m^–2^ s^–1^)	Ci (μmol CO_2_ mol^–1^ air)	Tr (mmol H_2_O m^–2^ s^–1^)	WUE (mmol CO_2_ mol^–1^ H_2_O)	AMC
W	17.13 ± 0.43^b^	0.31 ± 0.03^a^	237.77 ± 6.81^c^	5.46 ± 0.34^a^	3.14 ± 0.18^c^	0.07 ± 0.00^b^
WS	18.85 ± 1.25^a^	0.32 ± 0.02^a^	221.87 ± 4.42^c^	5.24 ± 0.31^a^	3.60 ± 0.19^a^	0.08 ± 0.01^a^
D	11.54 ± 0.67^d^	0.24 ± 0.01^b^	278.62 ± 3.96^a^	3.80 ± 0.03^b^	3.04 ± 0.20^c^	0.04 ± 0.00^d^
DS	13.38 ± 0.23^c^	0.25 ± 0.00^b^	258.61 ± 1.73^b^	4.97 ± 0.14^a^	3.37 ± 0.12^b^	0.05 ± 0.00^c^

Pn, net photosynthesis rate; Gs, stomatal conductivity; Ci, intercellular CO_2_ concentration; Tr, transpiration rate; WUE, water-use efficiency; AMC, apparent mesophyll conductance. Different lowercase letters indicate significant differences at P < 0.05.

### Transcriptomic responses in silicon-treated rice under dry cultivation show a unique pattern

**FIGURE 2:**
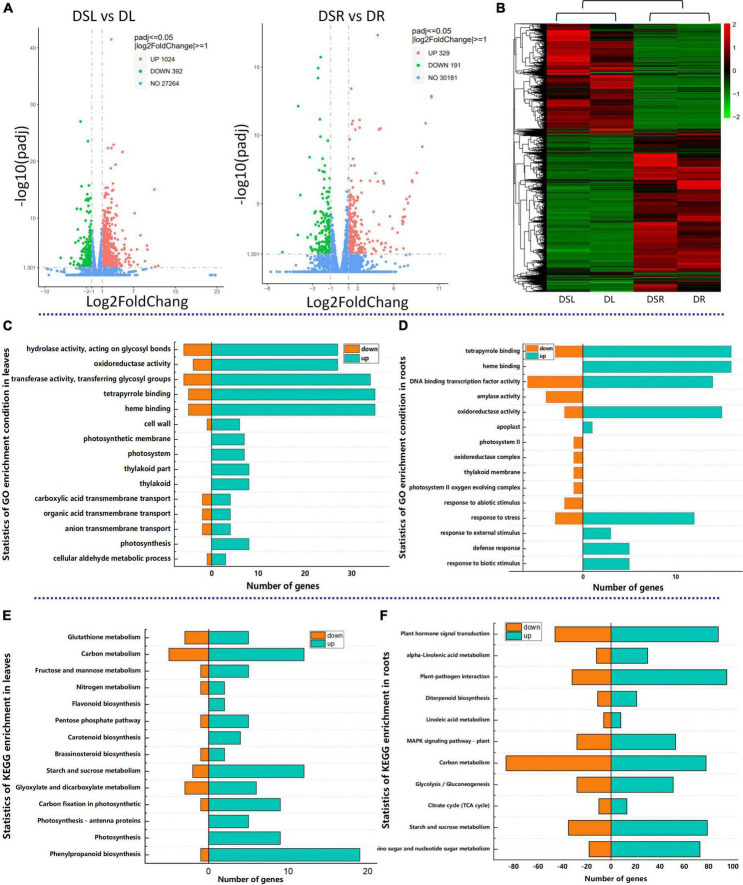
Analysis of effect of Si on the transcriptome of rice under dry cultivation. Volcano plots of differentially expressed genes (DEGs). DEGs in leaves (left) and roots (right). The horizontal coordinate shows the log2FoldChange value, and the vertical coordinate shows the –log10 *p*_*adj*_ or –log10 *p*-value. The blue dashed line indicates the threshold line for the differential gene screening criteria; Upregulated genes are in red, and downregulated genes are in blue **(A)**; Cluster dendrogram of differentially expressed genes in Si-treated and no Si-treated rice under dry cultivation **(B)**; GO enrichment with DEGs of leaves **(C)**; GO enrichment with DEGs of roots **(D)**; KEGG enrichment with DEGs of leaves **(E)**; KEGG enrichment with DEGs of roots **(F)**; The horizontal coordinate represents the ratio of the number of DEGs annotated in the GO/KEGG pathway, and the vertical coordinate represents the GO/KEGG pathway. DSL: Silicon-treated of rice under dry cultivation leaves; DL: Normal rice under dry cultivation leaves; DSR: Silicon-treated of rice under dry cultivation root; DR: Normal rice under dry cultivation roots.

Further, to dissect the differences in the transcripts of Si-treated rice under dry farming rice, an in-depth RNA-seq strategy was used to construct the global transcriptome profiles of the root and leaf tissues of dry farming rice grown with and without Si (X101SC21063706-Z01-J001-B1-16) (Supplementary Table 8). Samples were obtained at the 45 days after sowing under dry cultivation, these samples were used for the metabolome analysis also. The standards DESeq2 *p*_*adj*_ ≤ 0.05 | log2FoldChange| ≥ 1.0 were used to recognize the DEGs in response to drought. Analysis of the transcriptome data identified 520 DEGs in the roots of rice with Si application compared to those without Si application; 329 genes were upregulated, and 191 were downregulated. Similarly, 1416 DEGs were identified in the leaves of rice with Si application compared to those without Si; 1024 genes were upregulated and 392 were downregulated (Figure 2A). The samples were clustered together based on genes that showed similar expression patterns, the RNA-seq data were subjected to hierarchical clustering. Heatmap illustrating the Si-treated rice differed significantly from the no Si-treated rice under dry cultivation at the transcription levels also both in the roots and in the leaves (Figure 2B).

To gain insight into the biological processes of Si in response to drought stress, we analyzed the enrichment of gene ontology (GO) terms for DEGs. GO analysis revealed the top 10 enrichment categories for biological processes, cellular components, and molecular functions. The DEGs in the leaves and roots of dry farming rice treated with Si were enriched mainly in the biological process categories (Supplementary Table 1). We selected 15 GO enrichment processes for analysis. The significantly enriched GO terms of DEGs in DSL vs. DL were “oxidoreductase activity,” “hydrolase activity,” “tetrapyrrole binding,” “photosynthesis” and anion “transmembrane transport” (Figure 2C). The GO terms of DEGs in DSR vs. DR were enriched in “DNA binding transcription factor activity,” “amylase activity,” and “defense response,” except for “oxidoreductase activity” and “hydrolase activity” (Figure 2D).

Among the enriched GO terms, we observed that a large number of transcripts encoding transcription factors were differentially regulated after dry cultivation application of Si (570 transcription factors). The responsive transcription factors were grouped into several different families. These include WRKY, MYB, NAC, AP2 and bHLH, which play a key role in plant responses to abiotic stresses (Supplementary Table 2). In these families, the addition of Si, the larger part of these transcription factors in each cluster was upregulated.

The KEGG analysis revealed that most of the identified DEGs in the leaves were enriched in flavonoid biosynthesis, photosynthesis, carbon fixation in photosynthetic organisms and pentose phosphate pathway (Figure 2E), while in roots were enriched in amino sugar and nucleotide sugar metabolism, starch and sucrose metabolism, plant hormone signal transduction, and glutathione metabolism (Figure 2F). The results of GO and KEGG enrichment indicate that Si regulates various complex biological pathways of rice under dry cultivation.

### Metabolomic adjustment of rice treated with silicon under dry cultivation conditions

**FIGURE 3:**
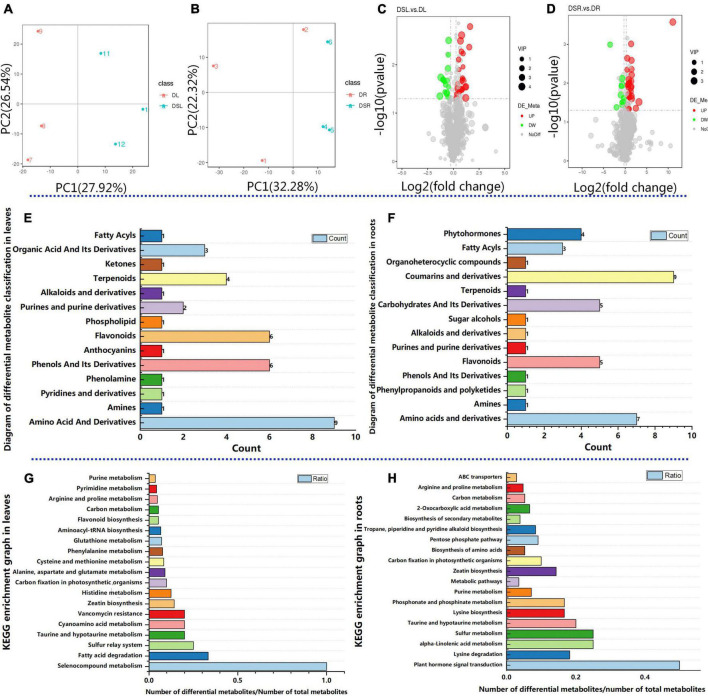
Effect of Si on quasi-targeted metabolomics in rice under dry cultivation. Principal component analysis (PCA) of metabolites in leaves **(A)** and roots **(B)**. The *X*-axis represents the first principal component (PC1), and the *Y*-axis represents the second principal component (PC2). Volcano plots for differentially accumulated metabolites (DAMs) from the leaves with and without Si treatment **(C)** and the roots with and without Si treatment **(D)**. Diagram of differential metabolite classification, with the horizontal coordinate showing the number of differential metabolites in the corresponding metabolic pathway and the vertical coordinate showing the corresponding metabolome classification in leaves **(E)** and roots **(F)**. Top KEGG enrichment results. The horizontal coordinate shows the number of differential metabolites in the corresponding metabolic pathway/the number of total metabolites identified in the pathway; the larger the value, the higher the enrichment of differential metabolites in the pathway in leaves **(G)** and roots **(H)**.

Further, to assess the metabolic changes in rice under drought, a quasi-targeted metabolome analysis was performed. The unsupervised hierarchical cluster analysis revealed a clear separation of the metabolites of rice with and without Si treatment (Figure 3A). Meanwhile, the PCA explained 54.46% of the total variance (27.92 and 26.54% for PC1 and PC2, respectively) in leaves, and 53.6% of the total variance (32.28 and 21.32% for PC1 and PC2, respectively) in roots. The PCA showed precise separation on PC1 between treatments with and without Si. The biological replicates were distinguished by PC2 (Figures 3A,B). These results indicated that differences in the metabolic reactions between rice plants with and without Si treatment form the basis for Si to alleviate the damage of rice under dry cultivation.

A total of 1100 metabolites were detected in the leaves and root (Supplementary Table 3). These known metabolites in leaves belonged to 61 classes, with the majority belonging to amino acid and derivatives, benzene and substituted derivatives, coumarins and derivatives, flavonoids, phenylpropanoids, carbohydrates and its derivatives, and phytohormones. Similarly, 61 classes of metabolites were identified in the root, with the majority belonging to Alkaloids and Amino Acid and Derivatives, Carbohydrates and its Derivatives, Coumarins and derivatives, and Flavonoids and Phytohormones (Supplementary Table 3). Interestingly, the DAMs associated with Phytohormones and Carbohydrates and its derivatives were not differentially accumulated in DSL vs. DL. However, the DAMs related to Amino acid metabolism, Organic Acid and its derivatives, and flavonoid biosynthesis were all highly accumulated in DSL vs. DL and DSR vs. DR. The results suggest that Si induce changes in metabolites in rice under dry cultivation. Differentially accumulated metabolites (DAMs) with a FC > 1.2, *P* < 0.05, and VIP > 1 were screened. Then, to identify DAMs related to the process of Si alleviating drought stress in dry farming rice, the metabolites in leaves and roots were compared. Out of the total 1100 metabolites, 38 DAMs (23 upregulated and 15 downregulated) were identified from the DSL vs. DL comparison (Figure 3C); these metabolites belonged to 14 classes in leaves (Figure 3E; Supplementary Table 4). Meanwhile, 41 DAMs (31 upregulated and 10 downregulated) were identified from DSR vs. DR (Figure 3D; these belonged to 14 classes in roots (Figure 3F; Supplementary Table 4).

Further KEGG pathway analysis revealed that the differential metabolites were enriched in different pathways. Differential metabolites identified in the leaves between with and without Si treatments were involved in the secondary metabolome, carbon fixation in photosynthetic organisms, phenylalanine metabolism, flavonoid biosynthesis, carbon metabolism, and amino acid metabolism (Figure 3G; Supplementary Table 5). Differential metabolites identified in the roots between with and without Si treatments were involved in plant hormone signal transduction, purine metabolism, carbon metabolism, amino acid metabolism, and biosynthesis of secondary metabolites (Figure 3H; Supplementary Table 5).

### Co-expression network analysis of differentially expressed genes and differentially accumulated metabolites in silicon-treated rice under dry cultivation

**FIGURE 4:**
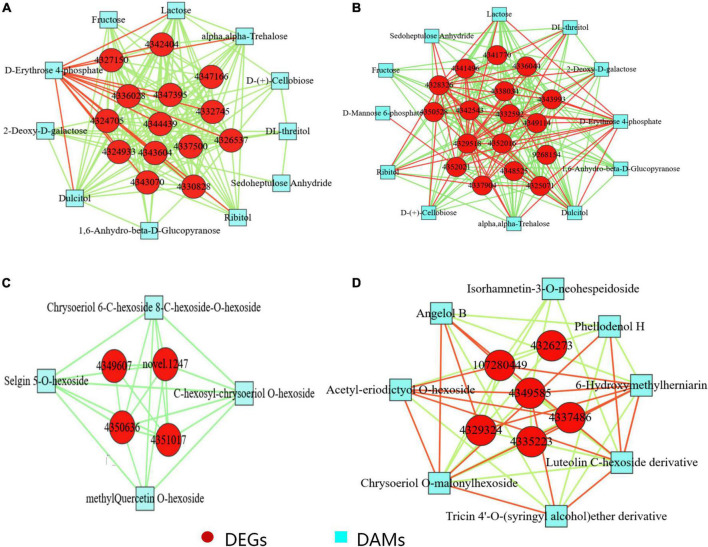
Co-expression analysis of DEGs and DAMs based on Pearson correlation coefficient. Interaction network of DEGs and DAMs involved in **(A)** photosynthesis in DSL vs. DL, 4326537, 4324933, 4337500, 4332745, 4327150, photosystem II gene; 4344439 ferredoxin gene; 4342404, 4347395, 4343070, 4330828, 4347166, photosystem I genes; 4343604, 4336028, 4324705, chlorophyll a-b binding protein. **(B)** Carbon metabolism in DSL vs. DL, 4329518, acetyl-coenzyme A synthetase; 4343993, malate dehydrogenase chloroplastic; 4325071, 4341770, fructose-bisphosphatase; 4341496, fructose-bisphosphate aldolase; 4350528, 6-phosphogluconate dehydrogenase; 4352021, 4328326, 4352016, ribulose bisphosphate carboxylase; 4342543, ribose-5-phosphate isomerase 4332592, acetate/butyrate–CoA ligase 4337904, acyl-coenzyme A oxidase; 4336044, glyceraldehyde-3-phosphate dehydrogenase; 9268154, malate synthase; 4349114, glycine cleavage system; 4348525, alanine aminotransferase 2; 4338034, 6-phosphofructokinase. **(C)** Flavonoid biosynthesis in DSL vs. DL, 4350636, novel.1247, chalcone synthase 1; 4349607, 4351017, chalcone–flavanone isomerase 3. **(D)** Flavonoid biosynthesis in DSR vs. DR. 4335223, cinnamyl alcohol dehydrogenase 6; 4349585, 4337486, cationic peroxidase; 4329324, cytochrome P450 CYP73A100; 107280449, 4326273, peroxidase. Red circles indicate differential genes, and blue squares indicate differential metabolites; Edges colored in “red” and “green” represent positive and negative correlations, respectively, based on Pearson correlation coefficient >0.8 and <–0.8, respectively.

Gene-metabolite interaction networks are used to understand the functional relationships and identify novel regulatory pathways. Here, the differentially expressed genes and metabolites associated with photosynthetic carbon fixation and flavonoid biosynthesis were tested for Pearson correlation under Si treatment conditions (Pearson correlation coefficient >0.8 or <−0.8; *P*-value < 0.05; Figure 4; Supplementary Table 6). A significant positive correlation was observed between the genes related to photosystem II reaction center protein, photosystem I reaction center subunit, chlorophyll a-b binding protein, ribose-5-phosphate isomerase, fructose-bisphosphatase, and ribulose bisphosphate carboxylase and metabolites, such as erythrose 4-phosphate, fructose, galactose, and sedoheptulose anhydride in DSL vs. DL. In DSL and DL, a different pattern of regulation was observed compared to the photosynthesis-related DEGs and DAMs, providing additional evidence that the efficiency of photosynthesis differed between the treatments.

To further analyze the impact of Si on the genes and metabolites related to secondary metabolism in rice under dry cultivation, the interaction of DEGs and DAMs related to flavonoid biosynthesis was analyzed. For flavonoid biosynthesis, Pearson correlation analysis was performed for four DEGs and four DAMs related to flavonoids biosynthesis in leaves (Figure 4; Supplementary Table 6). The DEGs showed strong positive correlation (*R*^2^ > 0.8 or <−0.8 and *P*-value < 0.05) with metabolites in leaves (Supplementary Table 6). For example, a significant positive correlation between the genes such as chalcone synthase 1 and chalcone–flavanone isomerase 3 and the metabolites such as chrysoeriol 6-C-hexoside 8-C-hexoside-O-hexoside, selgin 5-O-hexoside, C-hexosyl-chrysoeriol O-hexoside, and methyl quercetin O-hexoside in leaves. In roots, six DEGs related to flavonoids biosynthesis, cinnamyl alcohol dehydrogenase 6, and cytochrome P450 CYP73A100 were found to be upregulated in DSR. Moreover, the luteolin C-hexoside derivative, chrysoeriol O-malonylhexoside, acetyl-eriodictyol O-hexoside, and few coumarins and the downstream metabolites related to flavonoids biosynthesis were found to be accumulated in DSR.

### Quantitative real-time PCR validation of differentially expressed genes in silicon-treated rice under dry cultivation

**FIGURE 5:**
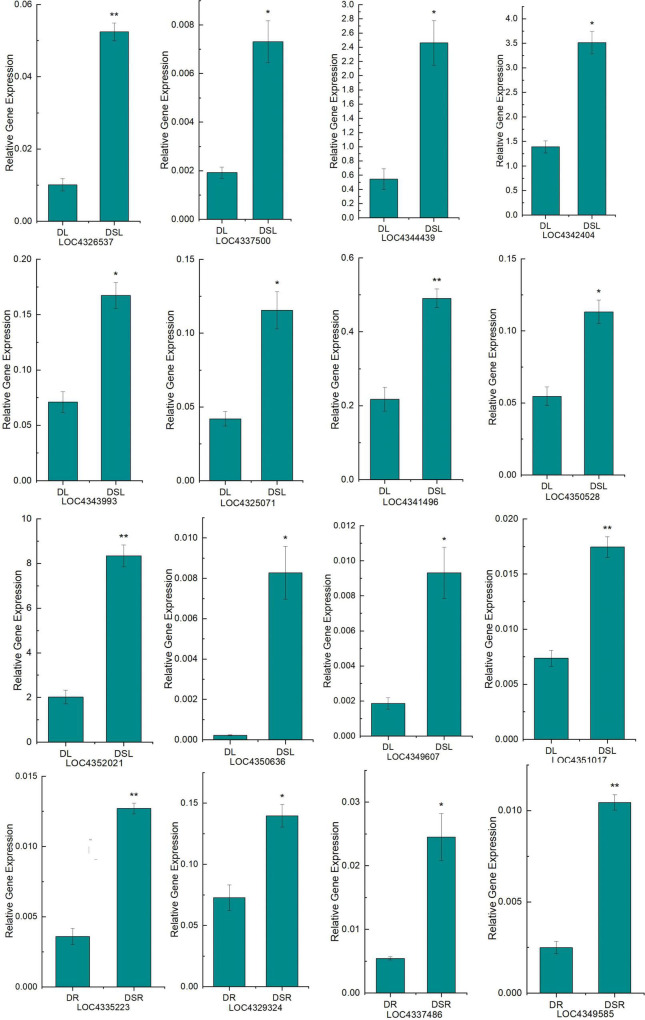
Quantitative real-time PCR (qRT-PCR) analysis of DEGs. The *X*-axis represents the treatment, and the *Y*-axis represents the relative expression of genes. DSL: Silicon-treated of rice under dry cultivation leaves; DL: Normal rice under dry cultivation leaves; DSR: Silicon-treated of rice under dry cultivation root; DR: Normal rice under dry cultivation roots. *P < 0.05, ^**^P < 0.01.

Finally, to validate our RNA-Seq data, quantitative RT-PCR (qRT-PCR) was performed on 16 DEGs (Figure 5; Supplementary Table 7) related to photosynthetic metabolism and flavonoid biosynthesis. These genes showed similar expression patterns in RNA-seq (FPKM) and qRT-PCR analyses, verifying the reproducibility and credibility of RNA-seq data.

## Discussion

### Positive role of silicon in rice under dry cultivation production

Dry cultivation of rice is a cropping pattern different from transplanted rice and helps save water and improve labor efficiency (Jiang et al., 2021). Rice under dry cultivation changes the living environment and is susceptible to biotic and abiotic stresses. Therefore, overcoming these limitations, improve rice adaptability under dry cultivation conditions and improving yields has been a major research topic. Silicon (Si) has been shown to play an important role in enhancing plant resistance to environmental stresses and improving plant growth and development (Ma et al., 2007; Ma and Yamaji, 2008). Usually, the Si could strengthen the membranes of plant cells by depositing around the cell walls and changing their permeability to various stresses such as salt and drought (Zhang et al., 2017). Chen et al. (2011) demonstrated that Si application to rice under drought stress increased total root length, root surface area, and biomass production. Nolla et al. (2012) and Ibrahim et al. (2018) reported that the application of Si to rice under drought stress increased plant height, biomass, and grain yield. The present study’s observations are consistent with most of these earlier results. Here, the application of Si had a significant effect on rice under dry cultivation; the root length, root volume and numbers were higher in the drought and flooding treatments with Si application than in the non-Si treatment. Additionally, applying Si in dry farming improved the yield (Su et al., 2022). The transcriptome and metabolome sequencing was performed to elucidate the mechanisms of Si regulation of adaptation in rice under dry cultivation. Under dry cultivation, Si treatment resulted in 520 DEGs in the root, including 329 upregulated and 191 downregulated ones, and 1416 DEGs in leaves, including 1024 upregulated and 392 downregulated ones. Moreover, 38 DAMs (23 upregulated and 15 downregulated) were identified from the DSL vs. DL comparison and 41 DAMs (31 upregulated and 10 downregulated) from the DSR vs. DR comparison. These observations indicate that Si could regulate the gene expression and metabolite accumulation of rice under dry cultivation. The effect of Si on the transcriptome and metabolome also has been investigated in several studies, such as under non-stress conditions, the effect of Si was minimal, a result confirmed in wheat and soybean (Watanabe et al., 2004; Rasoolizadeh et al., 2018). In contrast, the transcriptome recovered to control levels after adding Si under biotic and abiotic stress conditions (Zhu et al., 2019; Jonas et al., 2015). Supplementation of Si also affects gene expression levels. For example, Si alleviates stressful conditions in rice by regulating Cd transporter expression levels (Ma et al., 2015), downregulating jasmonic acid (JA) biosynthesis (Kim et al., 2014), downregulating OsZIP1 gene expression (Huang and Ma, 2020).

### Silicon improved the photosynthesis and carbon assimilation in rice under dry cultivation

**FIGURE 6:**
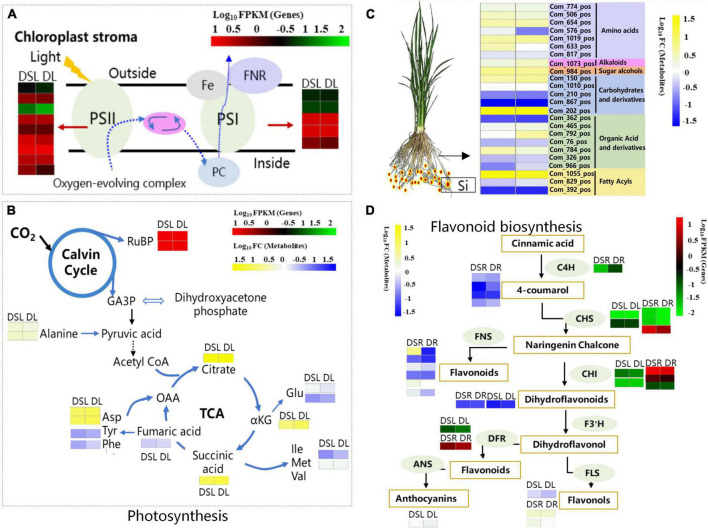
Summary model of metabolic processes regulated by Si on rice under dry cultivation. **(A)** Effect of Si on light reaction of rice leaves under dry cultivation. The DEGs in leaves and roots by the shade of red to green according to the color scale. **(B)** Effect of Si on the Calvin cycle of rice leaves under dry cultivation. The DEGs by the shade of red to green according to the color scale. The DAMs by the shade of yellow to blue according to the color scale. **(C)** Osmoregulation based on differential metabolites in roots. The DAMs by the shade of yellow to blue according to the color scale. **(D)** Flavonoid biosynthesis. The DEGs by the shade of red to green according to the color scale. The DAMs by the shade of yellow to blue according to the color scale in leaves and roots. C4H, cinnamic acid 4-hydroxylase; CHS, chalcone synthase; CHI, chalcone isomerase; F3′H, flavonoid 3′-hydroxylase; DFR, dihydroflavonol 4-reductase; ANS, leucoanthocyanidin dioxygenase; FLS, flavonol synthase.

Photosynthesis is one of the major metabolic pathways affected after silicon application; it is also the key physiological phenomena affected by drought stress (Farooq et al., 2009). During drought, the stomata close, reducing the availability of CO_2_ and making plants more susceptible to damage (Lawlor and Cornic, 2002). A similar phenomenon was observed under heat stress, which reduced the activity of photosystem II (Camejo et al., 2005) and impaired the regenerative capacity of RuBP (Wise et al., 2004). Ma X. et al. (2016) found that chloroplast genes showed similar expression patterns, providing evidence for different photosynthetic efficiencies in the two rice cultivars (Ma X. et al., 2016). Li et al. (2020) screened the transcriptomic data corresponding to photosynthesis pathway and found that the drought-responsive DEGs related to photosynthesis included those encoding PS II, PS I, and chlorophyll a/b-binding proteins. Meanwhile, nine PSII genes and eight PSI genes were downregulated under drought stress in rose leaves (Li et al., 2020). In this study, three chlorophyll a/b-binding protein genes, five PSII genes, five PSI genes, one ferredoxin gene, and two RuBP carboxylase gene were upregulated in rice under dry cultivation after Si treatment in DSL, while they were downregulated in DL (Figures 6A,B). Additionally, Si application increased the photosynthetic efficiency and enhanced the net photosynthetic capacity of rice under dry conditions (Table 1), this observation is consistent with the higher relative photosynthesis rate measured in DSL than DL. All these results showed that photosynthesis increased or remained good in the Si treatment, which reasonably explains why Si produced relatively high yields in the dry cultivation.

### Effect of silicon on osmoregulation in rice under dry cultivation

Osmoregulation is an important mechanism that allows plants to adapt to stress. Plants can survive longer and maintain metabolic processes in dry soil if osmoregulation occurs. Many substances, including inorganic cations and anions, organic acids, carbohydrates, and amino acids, contribute to osmoregulation (Sanders and Arndt, 2012). Ma X. et al. (2016) found 10 differential metabolites that significantly correlated with osmolality in drought-tolerant cultivar; osmotic adjustment improved drought tolerance in rice (Ma X. et al., 2016). In this study, 24 DAMs involved in amino acids and derivatives, carbohydrates and derivatives, organic acid, and fatty acyl were detected in DSR vs. DR (Figure 6C). In addition, the soluble sugar content of rice roots increased after Si application under dry cultivation (Figure 1). Soluble sugars play an important role in the osmoregulation of plants. In rice, Si maintains normal physiological and biochemical metabolism under dry conditions by regulating soluble sugars to increase the concentration of cytosol and reduce their osmotic potential. These results suggest that Si help maintain water uptake and cell turgor by accumulating large quantities of osmolytes, consequently maintaining the growth of rice under dry cultivation.

### Silicon improved the flavonoids biosynthesis in rice under dry cultivation

Flavonoids are a group of polyphenol compounds with antioxidant activities. Researchers have suggested that the accumulation of such substances could be a key step in regulating plant tolerance to different environmental stresses (Wang P. Q. et al., 2018; Chen et al., 2019). Gai et al. (2020) showed that exogenous ABA significantly affects the expression of genes involved in flavonoid biosynthesis; it also increased the content of flavonoids, anthocyanins, flavonols, and isoflavones and improved drought tolerance of tea (Gai et al., 2020). Raza et al. (2021) identified many DAMs closely associated with DEGs in various cold-tolerant oilseed rape varieties based on a combined transcriptome and metabolome analysis (Raza et al., 2021). Sun et al. (2021), based on transcriptome and metabolome analyses, revealed that DEGs were induced in both high and low phosphorus conditions in apple leaves and roots, and metabolome analysis revealed that the levels of amino acids and their derivatives, organic acids, and flavonoids were higher in roots under low P stress than in those under controlled growth conditions (Sun et al., 2021). Research has revealed that C4H, the second enzyme of the plant phenylpropanoid pathway, is involved in the drought defense of cucumber (Bellés et al., 2008) and the CHS from Abelmoschus esculentus regulated flavonoid accumulation and abiotic stress tolerance in transgenic Arabidopsis (Wang F. B. et al., 2018). A previous study also showed that the accumulation of quercetin and kaempferol under water deficit improved the drought tolerance of white clover (Lee et al., 2007). The main physiological functions of quercetin, kaempferol, luteolin C-hexoside derivative, and myricetin in plants were scavenging ROS and detoxifying free radicals (Yamasaki et al., 1997; Tattini et al., 2005; Iracema et al., 2007). Si application also upregulated the expression level of genes involved in flavonoid biosynthesis under drought stress (Ma D. Y. et al., 2014, 2016) thus protecting plants from drought affect. The present study found that C4H, CHS, CHI, DFR, and FLS genes were upregulated in DSL or DSR (Figure 6D). Meanwhile, three DAMs closely associated with differentially expressed genes (DEGs) were identified in DSL, and 13 DAMs, such as methyl quercetin, luteolin, angelol, and phellodenol, closely associated with DEGs in DSR. The accumulation of these flavonoids and derivatives is important during an environmental stress response. Flavonoids have been reported to play a role in the maintenance of ROS homeostasis (Chen et al., 2019; Mavrič Čermelj et al., 2021) also. In the present study, higher ROS levels (H_2_O_2_ and O_2_^–^ activity) were also detected in rice under dry cultivation compared with flooding treatment (Figure 1). Meanwhile, Si supplementation increased flavonoid-related gene expression and metabolite accumulation and reduced H_2_O_2_ and O_2_^–^ content in leaves. Moreover, to combat ROS attacks, plants recruit an impressive array of non-enzymatic and enzymatic antioxidants whose function is to maintain an adequate balance of ROS. Studies have shown that the application of Si can regulate ROS production during stress (Zhang et al., 2006) and increase these enzymatic activities in plants exposed to abiotic and biotic stresses (Liang et al., 2007). Ma D. Y. et al. (2016) found that Si application enhanced the expression of antioxidant enzyme coding genes in plants during drought stress which in turn help to reduce accumulation of H_2_O_2_ (Ma D. Y. et al., 2016). Silicon has significantly reduced O_2_^–^ and H_2_O_2_ (Figures 1E,F) by increasing SOD, POD, and CAT activities (Figure 1G) under both dry and flooding treatment. MDA and electrical conductivity serve as indicators of the extent of oxidative damage in stressed plants (Munns and Tester, 2008). Here, Si significantly reduced MDA content and electrical conductivity. As mentioned previously, ROS accumulation under dehydration decreases photosynthetic capacity (Chaves et al., 2009; Saibo et al., 2009). Si treatment improved ROS scavenging capacity, increased photosynthetic efficiency, and enhanced net photosynthetic capacity under dry conditions (Table 1). Therefore, we hypothesized that Si could regulate the adaptation of rice under dry cultivation by regulating the expression of flavonoid-related genes and accumulation of metabolites in the above-ground and below-ground parts to improve ROS scavenging ability, enhance membrane stability, and promote photosynthetic capacity and osmoregulation levels.

## Conclusion

The existing literature, reports of the benefits of silicon have proliferated of rice under flooded cultivation. Most of these studies typically focused on the impact of Si on drought, heat, cold, salt stress and metal toxicity and pathogens for recent reviews. However the role of Si in rice under dry cultivation has been little reported. The present study via physiological response combined with transcriptional and metabolic analyses found a positive influence of Si on aboveground and belowground parts rice under dry cultivation. Silicon is actively involved in physiological processes under dry cultivation and significantly improved photosynthetic performance and antioxidant enzyme activity and subsequently reduced lipid peroxidation of plant. Combined transcriptome and metabolome analyses revealed that Si upregulating flavonoid biosynthetic pathways in leaves and roots as well as osmoregulation in roots. These changes improved root parameters, nutrient uptake and transport, promoted root growth; while DEGs associated with photosynthesis were upregulated after Si treatment, enhancing photosynthesis and leading to increased organic matter production. Overall, these results emphasize that Si promotes the growth of rice under dry cultivation by coordinating the physiological metabolic processes of above- and belowground parts to enhance adaptation to stressful environments, and these findings also suggest that Si application is a feasible strategy to response to stress and will also promote further extension and application of rice under dry cultivation.

## Data availability statement

The datasets presented in this study can be found in online repositories. The names of the repository/repositories and accession number(s) can be found below: NCBI - PRJNA847389.

## Author contributions

X-SW and Z-HWu designed and supervised the project. HJ and X-SW analyzed the transcriptome metabolome data and wrote the manuscript. ZS, Q-WS, and Z-HWe participated in the determination of physiological indexes. W-CL and Z-XJ participated in the material preparation. PT, XY, Z-HWa, and M-YY revised the manuscript. Z-HWa analyzed the transcriptomic and metabolomic data. All authors discussed the results and commented on the manuscript.

## Footnotes

1
https://www.ricedata.cn/variety/varis/614593.htm

2
http://www.novogene.com

3
http://www.genome.jp/kegg/

4
http://www.hmdb.ca/

5
http://www.lipidmaps.org/

6
https://cloud.metware.cn/#/tools/tool-form?toolId=70

## Supplementary material

Supplementary Table 1 Gene ontology (GO) enrichment with DEGs of leaves and root in detail.

Supplementary Table 2 Details of leaf and root transcription factors in GO enrichment.

Supplementary Table 3 A total of 1100 metabolites were detected in the leaves and root, respectively.

Supplementary Table 4 All differential metabolites detected in leaves and roots.

Supplementary Table 5 Top KEGG enrichment results in leaves and roots in detail.

Supplementary Table 6 Co-expression analysis of DEGs and DAMs based on Pearson correlation coefficient associated with photosynthetic carbon fixation and flavonoid biosynthesis.

Supplementary Table 7 Primers used for quantitative RT-PCR analysis.

Supplementary Table 8 Original sequence read item.
